# Excess manganese increases photosynthetic activity via enhanced reducing center and antenna plasticity in *Chlorella vulgaris*

**DOI:** 10.1038/s41598-023-35895-x

**Published:** 2023-07-12

**Authors:** Amanda L. Smythers, Jessica R. Crislip, Danielle R. Slone, Brendin B. Flinn, Jeffrey E. Chaffins, Kristen A. Camp, Eli W. McFeeley, Derrick R. J. Kolling

**Affiliations:** 1 Department of Chemistry, Marshall University, Huntington, WV USA; 2 Present Address: Department of Chemistry, University of North Carolina at Chapel Hill, Chapel Hill, NC USA

**Keywords:** Bioenergetics, Antenna complex, Photosystem II

## Abstract

Photosynthesis relies on many easily oxidizable/reducible transition metals found in the metalloenzymes that make up much of the photosynthetic electron transport chain (ETC). One of these is manganese, an essential cofactor of photosystem II (PSII) and a component of the oxygen-evolving complex, the only biological entity capable of oxidizing water. Additionally, manganese is a cofactor in enzymatic antioxidants, notably the superoxide dismutases—which are localized to the chloroplastic membrane. However, unlike other metals found in the photosynthetic ETC, previous research has shown exposure to excess manganese enhances photosynthetic activity rather than diminishing it. In this study, the impact of PSII heterogeneity on overall performance was investigated using chlorophyll fluorescence, a rapid, non-invasive technique that probed for overall photosynthetic efficiency, reducing site activity, and antenna size and distribution. These measurements unveiled an enhanced plasticity of PSII following excess manganese exposure, in which overall performance and reducing center activity increased while antenna size and proportion of PSIIβ centers decreased. This enhanced activity suggests manganese may hold the key to improving photosynthetic efficiency beyond that which is observed in nature.

## Introduction

Once responsible for the largest extinction event known on earth, oxygenic photosynthesis now sustains most life through the generation of atmospheric oxygen, making it one of the most important biological mechanisms on the planet. Therefore it is of no surprise that photosynthetic metabolism has since evolved to be highly regulated and specific, enabling it to adapt to a variety of environmental stressors from the Archean eon to the modern age^1^. Of the most dangerous environmental stressors are those that result in the formation of reactive oxygen species (ROS), as the highly tuned electron transport mechanism relies on redox active metal cofactors to function effectively, including iron in cytochrome complexes *b*_559_ and *b*_6_*f*, iron-sulfur clusters in photosystem I (PSI) and ferredoxin, copper in plastocyanin, and manganese in the oxygen-evolving complex (OEC) of photosystem II (PSII)^2–5^. These metals have been evolutionarily ‘tuned’ to generate an average decreasing reduction potential throughout the photosynthetic electron transport chain (ETC), with electrons traveling energetically ‘downhill’ after being excited via photons at P680 in PSII until they reach plastocyanin. Then transferred to PSI, the electron is re-energized by another photon (via P700), allowing it to make the remaining electron transfers downhill again until it reaches ferredoxin reductase which then reduces nicotinamide adenine dinucleotide phosphate.

Environmental stressors, however, can disrupt this pathway due to either photoinhibition, wherein PSII is damaged via photooxidation and manganese ions are released from the OEC, or by the inhibition of PSII damage repair, which in turn disrupts the photoassembly of the OEC^6,7^. Additionally, these stressors often trigger the two modes of inhibition synergistically^8–10^. The exposure of the photosynthetic apparatus to metals, particularly redox-active transition metals, can result in an influx of damaging ROS, often caused by interactions with photosystems or their reaction intermediates. Metals including lead, cadmium, arsenic, and copper, among others, are known to inhibit photosynthesis and plant growth, decrease biosynthesis of pigment molecules, and decrease ETC phosphorylation, causing ETC dysregulation and dangerous ROS generation^11–14^. For example, increased copper exposure generates high levels of ROS, which then contribute to decreased chlorophyll biosynthesis, decreased photosynthetic efficiency and truncated growth, as well as structural and functional modifications to the light harvesting complexes (LHCs)^15–18^. Similarly, cadmium overexposure is associated with decreased cell yield, a severely decreased quantum efficiency, and the deactivation of non-photochemical quenching (NPQ), leading to increased photoexcitation and damage to the photosynthetic apparatus^19–21^. Iron is particularly damaging to photosystems as H_2_O_2_ and Fe(II) result in the Fenton reaction, producing ${\text{HO}}^{ \cdot }$ and${\text{HO}}_{{2}}^{ \cdot }$ , arguably the most biologically damaging ROS^22^. These ROS cause severe damage to proteins, lipids, and DNA, and are therefore implicated in the iron-dependent oxidative stress that induces an abundance of lipid radicals in *Chlorella vulgaris*^23^. Thus while both copper and iron are essential to ETC function, they are toxic when applied in excess concentrations due to ROS-mediated damage. However, this is not observed in photosynthetic organisms exposed to excess manganese; previous research has revealed that high manganese concentrations enhance photosynthetic capacity without causing intracellular damage^24^. This study seeks to determine the mechanism(s) through which this enhancement occurs.

Previous photosynthetic studies on manganese exposure utilized oxygen evolution and chlorophyll *a* (Chl *a*) OJIP fluorescence to determine the photosynthetic efficiency of *C. vulgaris* cells exposed to 17.5 and 35.0 mM manganese^24^. While F_V_/F_M_ is useful for determining the effect of stressors, it measures the average quantum efficiency of photosystems and does not provide insight concerning heterogeneity of the antennas or reducing site subpopulations of PSII. However, heterogeneity is critical in overall photosynthetic efficiency. The antennas of PSII are divided between PSIIα and PSIIβ reaction centers, where PSIIα refers to ‘super-complex’ light harvesting antennas with large antenna sizes and high degrees of connectivity located in the thylakoid grana and PSIIβ refers to ‘core complex’ light harvesting antennas with 2–3× smaller antennas and reduced connectivity located in the stroma-exposed region of the thylakoid^25,26^. The balance of these antenna systems determines the magnitude and efficiency of light absorption as well as the extent to which the inner chloroplast is shaded from sunlight. Simultaneously, PSII reaction centers exist as either Q_B_ reducing or Q_B_ non-reducing, the latter of which is incapable of contributing to the reduction of the plastoquinone pool and therefore do not contribute to overall electron flow^27^.

PSII heterogeneity is assessed through Chl fluorescence analysis, a widely-used non-invasive technique that provides fundamental information regarding the structure and function of PSII^2,28–30^. The rapid, highly sensitive, and non-destructive nature of Chl fluorescence makes it a useful technique for the analysis of biochemical stressors, including light stress, nutrient deprivation, and heavy metal contamination, among others^31–35^. In this study, we used a repertoire of Chl-analysis protocols in addition to oximetry to characterize the effects of elevated manganese concentrations on electron transfer efficiency, reducing site activity, and antenna heterogeneity of *C. vulgaris*. Based on our results, we propose that manganese enhances photosynthetic productivity in green algae and higher order plants and that excess manganese may facilitate protective mechanisms involved with photorepair and/or ROS scavenging. This suggests that manganese can be strategically used to increase the photosynthetic productivity of plant systems to enhance agricultural yield and/or carbon fixation.

## Results

### Photosynthetic performance and flux

**Figure 1:**
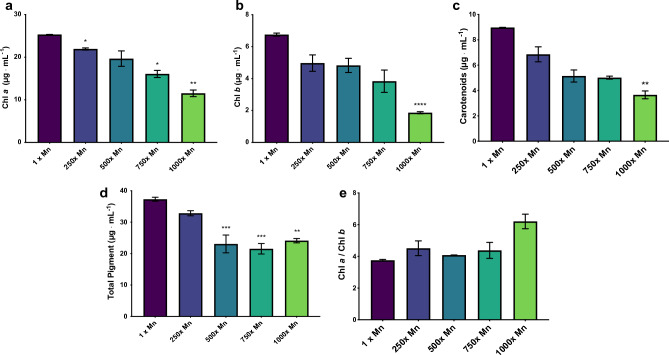
Pigment accumulation of *C. vulgaris* cultures exposed to increasing levels of manganese, divided into (**a**) Chl *a*, (**b**) Chl *b*, (**c**) carotenoids, (**d**) total pigment, and (**e**) ratio of Chl *a/*Chl *b*. All cells were measured in mid-exponential phase. The error bars represent standard error of the mean and statistical differences indicate a difference between the increased manganese and control cultures at one concentration. Significance determined by t-test is denoted by asterisks, where * indicates p ≤ 0.05, ** indicates p ≤ 0.01, *** indicates p ≤ 0.001, and **** indicates p ≤ 0.0001.

**Figure 2:**
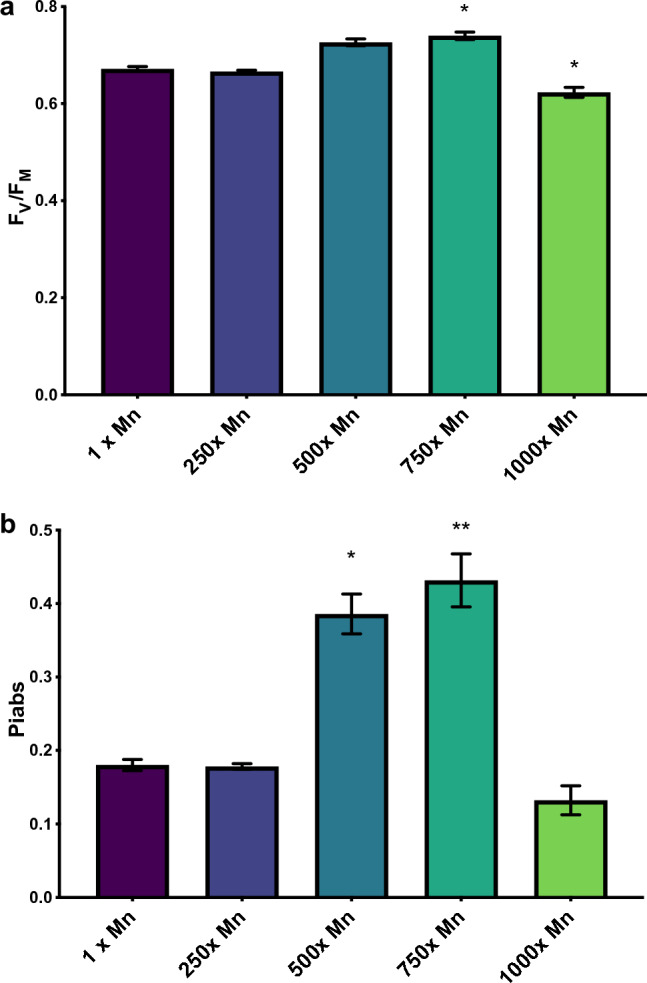
The photosynthetic performance of the *C. vulgaris* exposed to increasing concentrations of manganese. The error bars represent standard error of the mean and statistical differences indicate a difference between the increased manganese and control cultures at one concentration. Significance is denoted by asterisks, where * indicates p ≤ 0.05, ** indicates p ≤ 0.01, *** indicates p ≤ 0.001, and **** indicates p ≤ 0.0001. (**a**) The FV/FM, measured through OJIP fluorescence. FV/FM is the quantum yield of PSII in a dark-adapted state. (**b**) The PIabs of cultures, measured through OJIP fluorescence and calculated through the JIP test. PIabs quantifies the activity of PSII while simultaneously considering photon absorption, trapping of excitation energy, and conversion of energy to electron transport.

Previous work revealed that *C. vulgaris* accumulated manganese beyond equilibrium concentrations and that exposure to elevated levels of manganese resulted in an increased photosynthetic capacity over time as measured using F_V_/F_M_, the quantum yield of primary photochemistry^24^. This enhanced photosynthetic performance accompanied a significant increase in intracellular and adsorbed manganese, despite little to no effect on the overall turbidity. In order to determine the mechanisms responsible for this enhanced performance, cells were grown to mid-exponential phase—the point at which cells have the highest chlorophyll content and are most photosynthetically efficient—and measured at a concentration of 2.5 µg mL^−1^ total pigments to ensure the differing pigment content did not affect results (Fig. 1)^36,37^. It is essential to control for total chlorophyll prior to taking fluorescent measurements, as chlorophyll provides a proxy for total photosystem number, where lower amounts of chlorophyll is associated with lower numbers of PSII (Supplemental Fig. 1). Normalizing to chlorophyll analyzed ensures measurements assess the efficiency as opposed to the quantity of photosystems. F_V_/F_M_ was significantly increased and decreased in comparison to the control at 750 and 1000× manganese, respectively (Fig. 2a). More interesting, however, was the change in performance index on a per absorption basis (PIabs). As this parameter quantifies PSII activity normalized to total photon absorption, trapping of excitation energy, and conversion of energy to electron transport, it is a more accurate representation of photosynthetic activity^38,39^. The PIabs of the 500 and 750× manganese cultures were 2.1 and 2.4× higher than that of the control cultures (Fig. 2b). However, photosynthetic performance decreased dramatically in the 1000× manganese cultures, indicating that while increased manganese improves the photosynthetic performance to an extent, there is still a threshold at which overall activity is decreased.

**Figure 3:**
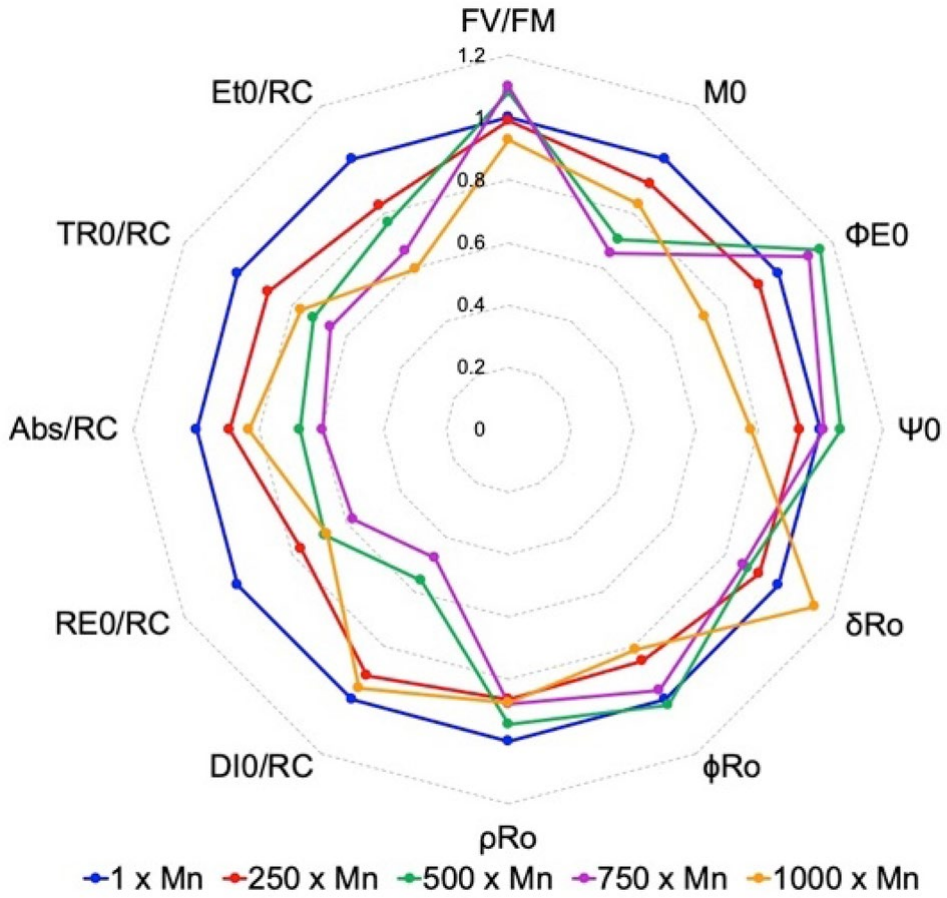
A spiderweb plot of JIP test parameters calculate from OJIP fluorescence measurements, in which the control cultures have been normalized to 1. For a review on JIP test parameters, see Stirbet et al.^87^.

In addition to an increasing performance index, exposure to increased manganese decreased the overall Chl fluorescence output. Both F_0_ and F_M_ decreased as manganese concentration increased, with F_M_ having an intensity of 1.755 ± 0.120 in the control cultures and an intensity of 1.128 ± 0.057 in the 1000× Mn cultures for a 36% depletion. These decreases could occur from either an increase in NPQ or an increased amount of active reducing reaction centers (or a combination of the two). However, the JIP test parameters indicate a more complex story (Fig. 3). All of the specific fluxes per reaction center decrease compared to the control, meaning the trapped energy (TR_0_/RC), electron transport flux (Et_0_/RC), dissipated energy flux (DI_0_/RC), and reduction of the acceptor side of PSI (RE_0_/RC) have all diminished. However, that is combined with an overall decrease in Abs/RC, the average absorbed photons per reaction center and an indicator of relative antenna size. Fewer photons would equate to less overall flux, but may increase the efficiency; in previously published work, *Chlorella* sp. mutants with truncated antenna complexes were more photosynthetically efficient, likely due to a reduction in shading and flux through NPQ^40,41^.

Another decreased flux is indicated by M_0_, the approximate initial slope of the OJ phase representing the rate of primary photochemistry to Q_A_^−^ (Fig. 3). M_0_ is inversely proportional to the rate of initial reaction center closure; thus, increased manganese slowed the rate of plastoquinone pool reduction. This was further supported by the increase in S_M_ across all manganese concentrations. S_M_ is the normalized area over the OJIP curve and is assumed to be proportional to the number of reductions and oxidations of one Q_A_ throughout the duration of the OJIP transient, allowing it to serve as an approximate number of electron carriers per ETC^42,43^. However, it is important to note that there is not a consensus in the correlation between S_M_ and the relative size of the reducing pool, as others have postulated that Q_A_ is fully reduced by the J step^44^. However, based on the summarized data, it appears that while photons absorbed per reaction center has seemingly decreased, the overall flux through the ETC has increased, therefore indicating an increase in active reaction centers.

### Reducing centers

**Figure 4:**
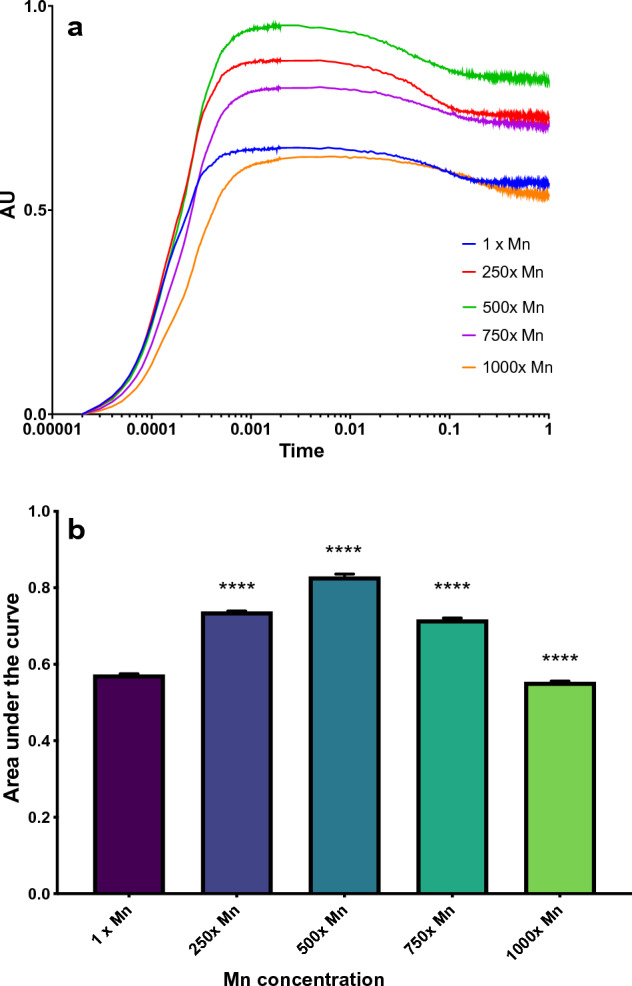
(**a**) The resulting Chl *a* transients following in vivo inhibition with DCMU. The curve is normalized where F_0_ = 0 and F_M_ = 1. The initial slope of the curve correlates to the time it takes to reduce the available Q_A_ centers. (**b**) The initial slope of the DCMU-inhibited Chl *a* transients. The error bars represent standard error of the mean and statistical differences indicate a difference between the increased manganese and control cultures at one concentration. Significance is denoted by asterisks, where * indicates p ≤ 0.05, ** indicates p ≤ 0.01, *** indicates p ≤ 0.001, and **** indicates p ≤ 0.0001.

**Figure 5:**
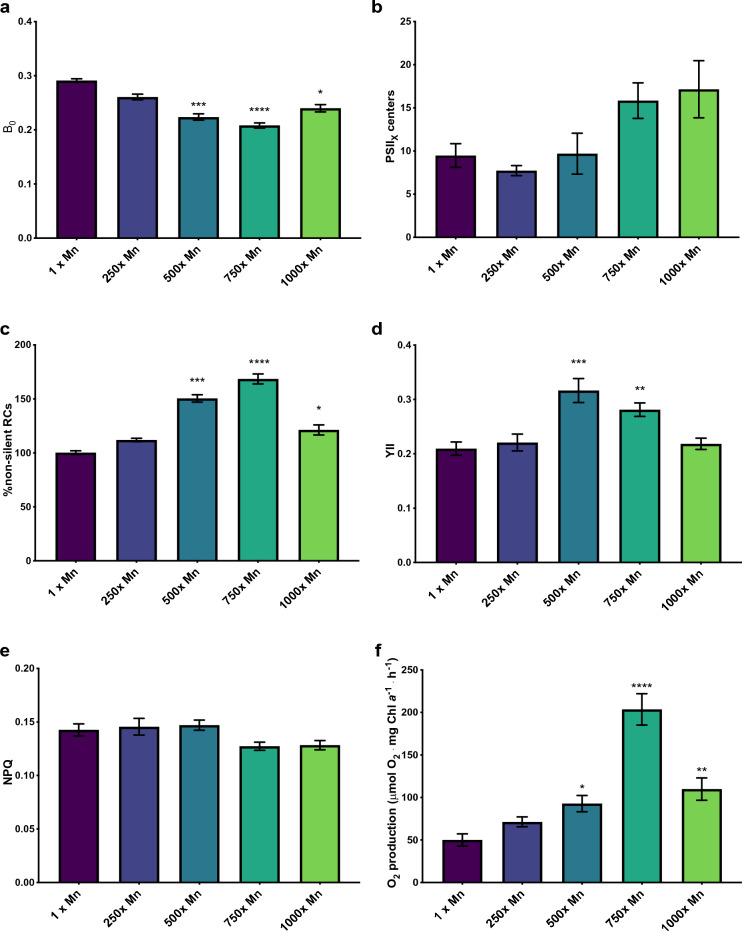
Measurements of PSII reducing activity. The error bars represent standard error of the mean and statistical differences indicate a difference between the increased manganese and control cultures at one concentration. Significance is denoted by asterisks, where * indicates p ≤ 0.05, ** indicates p ≤ 0.01, *** indicates p ≤ 0.001, and **** indicates p ≤ 0.0001. (**a**) The B_0_ of *C. vulgaris* exposed to increasing concentrations of manganese, indicating the quantity of Q_B_ non-reducing centers in the sample. (**b**) The PSII_X_ reaction centers as measured through difference in S4 of the S-state cycle. (**c**) The percent of non-silent reaction centers, calculated using parameters from the JIP test. (**d**) The flux of electrons through PSII, measured using pulse amplitude modulated fluorometry. (**e**) The relative amount of NPQ in cells exposed to increasing levels of manganese, measured using pulse amplitude modulated fluorometry. (**f**) The oxygen production of cells exposed to increasing concentration of manganese, measured using a Clark-type electrode.

Several fluorescence protocols enable the comparison of reaction center activity. In order to determine the relative amount of active Q_A_ sites, the initial point of electron transport in PSII, the cells were exposed to DCMU, an herbicide that selectively binds to the Q_B_ site of PSII and inhibits electron transfer^45–47^. The resulting fluorescent transient reaches its maximum output once all Q_A_ sites are reduced and does not allow for PSII turnover, generating a plot with an initial slope that inversely correlates to the relative amount of active Q_A_ sites within a sample (Fig. 4). Manganese appears to increase the activity of Q_A_ reducing centers, peaking at the 1000× manganese concentration with a 25% increase over the control. The relative number of non-reducing QB sites were determined using the double hit method for collecting the OJIP fluorescence transient. Similar to the Q_A_ reducing activity, the B_0_, or relative amount of non-reducing Q_B_ sites, decreases as the concentration of manganese increases, with peak activity occurring at 750× manganese (Fig. 5a). The 1000× manganese cultures are also less active compared to the 500× and 750× manganese cultures, but, unlike the Q_A_ sites, do not surpass the control cultures, demonstrating an increased activity of Q_B_ even at 70 mM manganese.

In order to understand the full effect of Q_A_ and Q_B_ activity, it is necessary to comprehensively measure the activity of PSII reaction centers. Previous studies have accomplished this through measurements of S-state populations, wherein the fluorescence decay after the fourth flash is controlled almost entirely by PSII_X_^48–50^. When measured using the difference between S_4_ and S_0_ of the S-state cycle, it would appear that PSII_X_ (relative inactive PSII) were increasing, despite noted increases to reducing center activity (Fig. 5b). However, these differences did not show statistical significance, a problem described in a previous study of *C. vulgaris* exposed to excess lead^32^. When using the OJIP method to determine the percent of non-silent reaction centers (Eq. 5), all concentrations 500× and over increased in overall activity, with the 750× manganese showing a peak activity 68% higher than that of the control (Fig. 5c). While the 1000× manganese cultures revealed a significantly increased percent of silent reaction centers when compared to the 750× Mn, this concentration still possessed a higher activity that the control, thus showing a correlation between manganese and increased PSII activity. The enhanced reaction center activity of PSII is further corroborated using PAM fluorescence, through which electron flux through PSII (YII) were significantly increased at both 500 and 750× manganese (Fig. 5d), indicating that absorbed photons are being increasingly utilized to propel photosynthetic electron transfer rather than dissipated via NPQ and/or fluorescence under excess manganese exposure. This was also observed through measuring oxygen production, which showed significant increases at 500, 750, and 1000× manganese (Fig. 5f). These measurements were taken at high light intensity in order to ensure for total PSII saturation, which correlates oxygen production to the number of total active PSII in a given sample. Thus the increase in oxygen likely correlates to more active PSII upon exposure to increased manganese, thus suggesting that excess manganese contributes to a healthier and more active ETC. Therefore, despite the discrepancy revealed through S-state cycle measurements, the data supports an overall increased activity of PSII reaction centers when *C. vulgaris* is grown in elevated manganese concentrations.

### Antenna complexes

**Figure 6:**
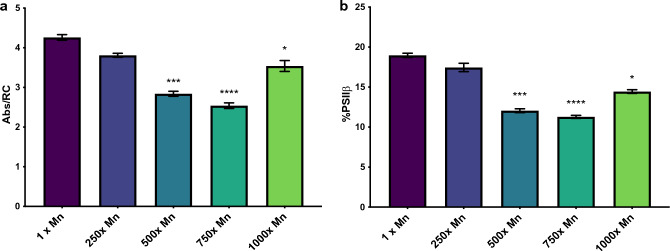
Measurements of relative antenna size in *C. vulgaris* exposed to increasing concentrations of manganese. The error bars represent standard error of the mean and statistical differences indicate a difference between the increased manganese and control cultures at one concentration. Significance is denoted by asterisks, where * indicates p ≤ 0.05, ** indicates p ≤ 0.01, *** indicates p ≤ 0.001, and **** indicates p ≤ 0.0001. (**a**) The absorption of chlorophyll antennae on a per reaction basis, indicating the relative antenna size and measured through OJIP fluorescence. (**b**) The percent of PSIIβ centers as measured through flash fluorescence induction.

Increased photosynthetic efficiency could be dependent on the activity of PSII reaction centers as well as the absorptivity of antenna complexes. Antenna complexes are measured through Chl *a* fluorescence induction, which calculates the average photons absorbed per reaction center (ABS/RC) to approximate the apparent antenna size. Larger antennae will increase absorption capacity for photons, thus increasing ABS/RC. As the concentration of manganese increased, the ABS/RC decreased, reaching its plateau at 750× manganese with a 40% decrease in size compared to the control and suggesting an inversely proportional relationship between manganese concentration and antenna size (Fig. 6a). However, PSII antennas are not stagnant: antenna complexes are dynamic and fluctuate in size and position as PSIIα often converts to the smaller PSIIβ under stress conditions and the antenna complexes shift between state 2 and state 1. Thus, a decrease in antenna size could be the result of these phenomena. To determine the relative proportion of PSIIα to PSIIβ, we used fast fluorescence induction (FFI), which implements a single turnover saturating flash to induce rapid Q_A_ reduction^51^. Interestingly, FFI revealed an overall decrease in PSIIβ centers (Fig. 6b). This indicates that although Chl content and ABS/RC are both diminished in cultures exposed to excess manganese, they are still primarily relying on PSIIα supercomplexes, therefore suggesting that the decreased antenna size is from a decreased number of antennas and associated reactions centers rather than interconversion to smaller complexes.

To further analyze the manganese-induced changes to the antenna complexes, low temperature (77 K) fluorescence spectroscopy was used to assess the distribution between LHCI and LHCII and its dependence on manganese concentration. Low temperature fluorescence results in a dual peaked trace with maxima at 685 nm and 730 nm, and has been used to distinguish the presence of state 1 and state 2 transitions in vivo*,* through which state 1 is shown through a shift toward lower wavelengths^52^. These transitions enable a more finely tuned balance of light absorption by the photosystems and allow rapid acclimation to changes in light intensity, making state transitions a key component of photoprotection^53,54^. Previous research has isolated the component parts of the photosynthetic cores and LHCs in order to distinguish their contributions to the overall spectra^55–59^. Thus, by assuming a non-linear Gaussian distribution and aligning to the best fit, the component peaks representing LHCII (681 nm), CP47/CP43 (686.5 nm), CP47 (695.5 nm), PSI core (721.5 nm), and LHCI (735 nm) could be individually assessed (Table 1, Supplemental Fig. 2). This revealed a decrease in PSII-associated LHCII at the 250×, 500×, and 750× manganese concentrations, mirroring that which was described from the decreasing ABS/RC and correlating manganese with decreased antenna size. By taking the ratio of the LHCII:LHCI peaks, it is possible to delineate relative number of antennas associated with PSII compared to PSI, which can suggest the prominence of PSII-initiated linear electron flow relative to PSI-initiated cyclic electron transfer. As the concentration of Mn increases, the LHCII:LHCI ratio increases, with significantly increased ratios at 250× (p = 0.02) and 500× (p = 0.01) manganese. The rising LHCII:LHCI ratio demonstrates that manganese either promotes an ETC that is increasingly reliant on PSI-initiated light harvesting, or that that *C. vulgaris* grown in excess manganese exhibits fewer LHCII antenna complexes. The latter is more likely as the cultures with increased manganese had lower levels of pigment accumulation (Fig. 1) and the aforementioned decreased ABS/RC (Fig. 6a). This is complicated by the decrease in PSIIβ centers (Fig. 6b); however, since multiple photosystems can receive photons from one supercomplex, it is possible that fewer overall LHCs are needed in the higher Mn cultures.

**Table 1:** The integration (μ) of the peaks deconvoluted from the 77 -K spectra (n = 4) and the potential deviation from the model (δ).

λ	1 × Mn		250 × Mn		500 × Mn		750 × Mn		1000 × Mn	
	μ	δ	μ	δ	μ	δ	μ	δ	μ	δ
681	8.85 × 10^4^ ± 16%	7.72 × 10^3^ ± 9%	5.96 × 10^4^ ± 24%	8.93 × 10^3^ ± 12%	6.97 × 10^4^ ± 21%	8.54 × 10^3^ ± 13%	6.29 × 10^4^ ± 2%	5.71 × 10^3^ ± 26%	1.04 × 10^5^ ± 30%	9.23 × 10^3^ ± 25%
686.5	1.07 × 10^5^ ± 9%	8.99 × 10^3^ ± 8%	1.45 × 10^5^ ± 14%	1.09 × 10^4^ ± 11%	1.46 × 10^5^ ± 18%	1.01 × 10^4^ ± 13%	1.04 × 10^5^ ± 26%	6.62 × 10^3^ ± 27%	1.52 × 10^5^ ± 31%	1.06 × 10^4^ ± 25%
695.5	1.37 × 10^5^ ± 11%	4.21 × 10^3^ ± 11%	1.33 × 10^5^ ± 10%	4.59 × 10^3^ ± 10%	1.52 × 10^5^ ± 18%	4.30 × 10^3^ ± 13%	1.19 × 10^5^ ± 26%	2.82 × 10^3^ ± 31%	1.68 × 10^5^ ± 23%	4.56 × 10^3^ ± 16%
721.5	7.64 × 10^5^ ± 9%	1.11 × 10^4^ ± 18%	1.02 × 10^6^ ± 13%	1.29 × 10^4^ ± 15%	9.94 × 10^5^ ± 18%	1.20 × 10^4^ ± 18%	6.27 × 10^5^ ± 23%	7.75 × 10^3^ ± 34%	9.86 × 10^5^ ± 34%	1.35 × 10^4^ ± 22%
735	4.65 × 10^5^ ± 8%	1.70 × 10^4^ ± 14%	5.23 × 10^5^ ± 10%	2.24 × 10^3^ ± 12%	5.45 × 10^5^ ± 16%	1.84 × 10^4^ ± 18%	3.78 × 10^5^ ± 25%	1.32 × 10^4^ ± 33%	5.59 × 10^5^ ± 22%	2.16 × 10^4^ ± 19%
LHCI:LHCII	5.32 ± 11.9%		9.17 ± 26.9%		7.97 ± 19.1%		5.97 ± 11.3%		5.46 ± 8.31%	

Relative standard deviation is denoted by percent.

## Discussion

While photosynthesis and light harvesting relies on several important metal cofactors, excess exposure to many of them—including copper, iron, and magnesium—leads to disruption of the photosynthetic ETC^60,61^. Our results indicate that this is not the case with manganese: in fact, overall performance increases with increasing manganese concentrations (Fig. 2b). Based on fluorescence measurements, this is facilitated by an increase in the activity of reaction centers paired with a decrease in PSIIβ complexes and decrease in average antenna size (Figs. 5, 6). This increased activity could result from an increase in manganese concentration within the chloroplast itself. While determining subcellular localization was outside the scope of this study, previous studies in algae have shown a tendency for metals to accumulate both in cell walls as well as in plastids^62–64^. Furthermore, our previous work determined that *C. vulgaris* accumulates intracellular manganese up to 55× the surrounding medium, suggesting that these manganese stores may be compartmentalized into an organelle or complexed in order to vastly exceed equilibrium concentrations^24^. Chloroplastic storage could be facilitated by plastidal manganese transporters. *Chlamydomonas reinhardtii*, a model green alga, encodes two distinct chloroplastic manganese transporters via *PAM71-HL* and *CGLD1*^65–67^. However, these proteins are not found in the *Chlorella* genome; a BLAST search resulted in no matches over a 50% similarity threshold. Unfortunately, the limited genetic annotation of *C. vulgaris* prohibits thorough understanding of the genetic and proteomic regulation that could increase manganese stores outside of equilibrium.

OEC assembly is not facilitated by protein chaperones, but requires a redox potential similar to that of Chl *a*, enabling the initial Mn^2+^ to Mn^3+^ oxidation to occur on the surface of PSII^68^. Thus enhanced manganese concentration in the chloroplast could increase the accessibility of manganese for PSII binding sites, and push the reaction kinetics forward toward more efficient de novo OEC synthesis, thus increasing the rate of photoassembly. These increases in photoassembly efficiency may then contribute to the observed performance increases by enabling more efficient rate of photorepair following the turnover of PSII D1 dimers^69,70^. Photoinhibition and the subsequent photorepair cycle is inevitable, and the overall efficiency of the photosynthetic apparatus is dependent on the difference between the rate of photoinhibited and photorepaired PSII^71^. Increasing the efficiency of this photorepair would therefore generate a net increase in active PSII units compared to basal conditions, potentially increasing the overall performance and resulting in the increased activity observed herein. This is supported by studies that have shown manganese-deficient plants to decrease in photosynthetic efficiency due to PSII structural instability, with the magnitude of instability related to manganese binding affinity to PSII and suggesting that photorepair is, in part, concentration dependent^72^. However, since PSII repair requires the translation of D1 subunits directly into the membrane, it is likely that the synthesis and insertion of D1 is rate-limiting^68^. Therefore, it is likely that increased manganese concentration has a threshold for increasing repair efficiency, as shown here through the decreased photosynthetic flux at the higher concentrations of manganese.

The likelihood that photorepair efficiency has improved is further supported by the decrease in PSIIβ complexes. Previous research has determined that PSIIβ complexes are localized to the stroma lamellae of the chloroplast, in which damaged PSII monomers are repaired and new PSII units are synthesized^73–75^. Thus a decreasing amount of PSIIβ could indicate a faster flux out of the stroma lamellae. The decreased PSII-bound LHCII complexes in the higher Mn cultures as revealed by the low temperature fluorescence supports this further, as complexes could more easily share LHCII super complexes if there is less flux toward PSII repair. However, this switch to a predominantly LHCI-based system could also be an artifact of the enhanced PSII activity. If the flux of electrons through PSII increases due to increasing reducing center activity, the plastoquinone pool will then become oxidized faster. This would then increase the flow of electrons toward the downstream proteins, potentially enhancing the turnover rate of PSI. Ergo the switch to PSI-bound LHCI may be generated from the increased rate of oxidation at the acceptor side of PSII as the entire photosynthetic electron transport chain moves electrons at a faster overall flux.

Another possibility is that the manganese is helping protect the photosynthetic ETC from sustaining damage in the first place, which may explain why the level of YII increases while the NPQ remains unchanged across concentrations (Fig. 5d, e). While unexplored in algae, manganese has been shown to increase oxidative stress resistance in organisms with active superoxide dismutase enzymes in a manner dependent on high manganese/iron ratios within the cell, allowing such a boost in antioxidant capacity that it helped facilitate resistance to gamma-radiation^76–78^. Furthermore, it was shown that this manganese-facilitated resistance protected proteins, not DNA; thus, manganese may have a role in protecting proteinaceous components of the photosynthetic ETC from oxidation in *C. vulgaris*^79^. Additionally, adding excess manganese to the growth media of yeast with lethal superoxide dismutase knockouts rescued the phenotype, allowing growth under aerobic conditions and increasing scavenging activity with no change in enzymatic catalase or peroxidase activities^80^. Manganese is also capable of catalase-like activity and can disproportionate H_2_O_2_ in the presence of bicarbonate, independent of enzymatic processes^81^. Furthermore, manganese can form non-proteinaceous complexes through mixtures of phosphate, peptides, carbohydrates, and nucleoside bases, with which it can actively scavenge superoxide radicals^82–84^. Manganous phosphate salts are particularly effective, as they rapidly catalyze the scavenging of superoxide radicals using a mechanism completely distinct from that of superoxide dismutases, in which Mn^2+^ reacts to form a short-lived MnO^2+^ before disproportionating to form manganese phosphate, dioxygen, and hydrogen peroxide^85^. Further studies are needed to determine if increased reducing activity is due to an increase in photorepair efficiency, a decrease in photoinhibition, or a combination of the two.

## Materials and methods

### Strain and culture growth conditions

Cultures of *C. vulgaris* (Carolina Biological Supply) were maintained on lysogeny broth agar plates and for batch cultures, inoculated into 25 mL of modified *Chlorella* medium supplemented with 20 g L^−1^ dextrose. All cultures were grown in 50-mL sterile Erlenmeyer flasks capped with aluminum foil^37^. Cultures were grown in triplicate, using a 1-mL inoculum from a stationary phase culture and kept under constant white light at 30 µmol photons m^−2^ s^−1^ via an LED light at 25 °C with an orbital rotational speed of 100 rpm (verified using a tachometer).

### Experimental design

Prior to culture inoculation, manganese-deprived modified *Chlorella* medium (containing 20 g L^−1^ dextrose) was dosed with an autoclaved stock of MnCl_2_ and ultrapure H_2_O, ensuring that all cultures had equal concentrations of all other medium components. The control cultures’ media was dosed to generate a working concentration of 0.070 mM MnCl_2_, the standard concentration for modified *Chlorella* medium^37^. Experimental cultures’ media were 250× (17.5 mM) to 1000× (70.0 mM) the control with an n = 6. Cultures were maintained under constant light and grown until they reached mid-exponential phase, shown through a spectroscopic cell density of between 8 and 10 AU and peak pigment concentration for that particular experimental group. Mid-exponential phase was chosen to assay each culture at its peak photosynthetic productivity.

### Spectroscopic cell density

Cell density (turbidity) was obtained using a Shimadzu UV-1800 spectrophotometer (Shimadzu Corp., Kyoto, JP) at 750 nm as previously described^37,86^.

### Pigment extraction

Pigments were extracted using DMSO as previously described and measured from 470 to 700 nm^37^. Chl *a,* chlorophyll *b* (Chl *b*), and total carotenoids were calculated using the following equations^86^:

1 $$ \left[ {{\text{Chl}}a} \right] = \left( {{12}.{47 } \times {\text{ Abs}}_{{{665}.{1}}} } \right){-}\left( {{3}.{62 } \times {\text{ Abs}}_{{{649}.{1}}} } \right) $$

2 $$ \left[ {{\text{Chl}}b} \right] = \left( {{25}.0{6 } \times {\text{ Abs}}_{{{649}.{1}}} } \right){-}\left( {{6}.{5 } \times {\text{ Abs}}_{{{665}.{1}}} } \right) $$

3 $$ \left[ {{\text{Carotenoids}}} \right] = \, {{\left[ {\left( {{1}000 \, \times {\text{ Abs}}_{{{48}0}} } \right){-}\left( {{1}.{29 } \times \, \left[ {{\text{Chl}}a} \right]} \right){-}\left( {{53}.{78 } \times \, \left[ {{\text{Chl}}b} \right]} \right)} \right]} \mathord{\left/ {\vphantom {{\left[ {\left( {{1}000 \, \times {\text{ Abs}}_{{{48}0}} } \right){-}\left( {{1}.{29 } \times \, \left[ {{\text{Chl}}a} \right]} \right){-}\left( {{53}.{78 } \times \, \left[ {{\text{Chl}}b} \right]} \right)} \right]} {{22}0}}} \right. \kern-0pt} {{22}0}} $$

### Photosynthetic electron transfer fluxes

Photosynthetic electron transfer fluxes were inferred from in vivo Chl *a* fluorescence using a Photon Systems Instruments FL 3500 fluorometer following a 5 min dark adaptation as previously described^36^. To ensure reproducibility between samples, algal cultures were diluted to 2.5 µg mL^−1^ total pigments using medium of the same manganese concentration before dark adaptation and subsequent measurements. The OJIP protocol included a 1-s actinic illumination using a 630-nm light at an intensity of 2400 µmol photons m^−2^ s^−1^. Fluorometry parameters (JIP test) were calculated as previously outlined (Stirbet et al.^87^).

### Determination of reducing center activity

To further increase understanding of the mechanisms at work and to estimate the relative amount of Q_B_ non-reducing centers, the double hit method was used, collecting Chl *a* fluorescence data at two subsequent 1-s pulses^32,34,88^. This method generates sequential OJIP transients that can be separated and normalized to t = 0. While the first pulse was conducted following dark adaptation, meaning that all the reaction centers were open, the second pulse will only excite so-called ‘fast-opening’ reaction centers, allowing for the calculation of non-reducing centers (centers which are unable to open in time for the second pulse) using the equation:

4 $$ {\text{B}}_{0} = \, \left( {{\text{F}}_{{\text{V}}} /{\text{F}}_{{\text{M}}} {-}{\text{ F}}_{{\text{V}}} */{\text{F}}_{{\text{M}}} *} \right)/{\text{F}}_{{\text{V}}} /{\text{F}}_{{\text{M}}} $$

where F_V_/F_M_ is derived from the first pulse and F_V_*/F_M_* is derived from the second pulse.

The redox state of the OEC can be determined using short, actinic light flashes to sequentially advance from S0 to S4, where each S-state represents a progressively more oxidized state of the OEC^89,90^. The contribution of inactive PSII (PSII_X_) centers can be estimated by the difference between the S4 S0 fluorescence decay and the initial F_0_, since the fluorescence decay following the fourth flash is primarily controlled by inactive centers^48,91^. S-states were measured through 4 saturating flashes at 80,000 µmol photons m^−2^ s^−1^ voltage that were 100 µs in duration and 300 ms apart, each causing a single turnover of PSII^32^. Cells were dark adapted for 5 min prior to measurements.

In addition to quantifying inactive Q_B_ centers, active Q_A_ reducing centers were quantified through the inhibition of PSII with 3-(3,4-dichlorophenyl)-1,1-dimethylurea (DCMU), a selective inhibitor that blocks the Q_B_ site^92–94^. When DCMU-inhibited cells are measured for Chl *a* fluorescence, the normal polyphasic transient is replaced by a single-phase plateau, indicating the single turnover of Q_A_ to Q_A_^-^. By comparing the slope of the normalized curve when t = 300 μs, it is possible to infer the relative number of active reducing centers, as a decreased slope would indicate a longer time needed to reduce all Q_A_, therefore suggesting an overall increase in activity^39^. Dark adapted samples were exposed to 50 mM DCMU prepared in DMSO for 10 min before measuring using the same protocol used for the JIP test. DCMU was prepared as a 1.5 M stock and combined with cell media to form a 1 mL solution to maintain DMSO concentrations below 5% and prevent damage to photosynthetic proteins. GraphPad Prism v.7.01 was used to perform the integrations following normalization^32^.

It is also possible to calculate active Q_A_ centers using JIP test parameters, in which the absorption flux per reaction center (ABS/RC) is used in the following equation^30,32,95,96^:

5 $$ {\text{RC}}^{{{\text{si}}}} = \left[ {1 - { }\frac{{\frac{{{\text{ABS}}}}{{{\text{RC}}}}{\text{control}}}}{{\frac{{{\text{ABS}}}}{{{\text{RC}}}} \uparrow {\text{Mn}}^{2 + } }}{ }} \right] \times 100 $$

For control (0.07 mM manganese) cultures, the average ABS/RC of all cultures was used as the control, in order to determine the overall standard error in activity. In order to report increases in activity, the RC^si^ value was multiplied by − 1 and added to 100, resulting in a measurement of percent activity as compared to the control condition.

Last, the size of the reducing pool can be estimated by calculating the normalized complimentary area over the OJIP curve; since this area is assumed to be proportional to the number of reductions and oxidations of one Q_A_ center, it enables relative comparison of the number of electron carriers along the ETC^39,97^. To calculate the normalized area, the OJIP was double normalized where F_O_ = 0 and F_P_ = 1 before using GraphPad Prism v.7.01 to calculate the complementary area above the curve. To allow comparisons between experimental groups, the area was divided by the variable fluorescence.

### Non-photochemical quenching analysis

Quenching analysis was used on dark-adapted cells with an actinic intensity of 300 µmol photons m^−2^ s^−1^, a saturating pulse intensity of 64,000 µmol photons m^−2^ s^−1^, and a measuring flash voltage of 80%. There was a dark relaxation duration of 20 s between pulses^98^. Photochemical coefficients were calculated as previously reported^39^.

### Antenna size and heterogeneity

Antenna size was estimated by the calculation of Abs/RC by the following equation^33,39^:

6 $$ \frac{{{\text{ABS}}}}{{{\text{RC}}}} = \frac{{4\left( {{\text{F}}_{{300{\text{us}}}} - {\text{F}}_{0} } \right)}}{{{\text{F}}_{{\text{V}}} }}{ } \times { }\frac{1}{{{\text{F}}_{{2{\text{ms}}}} - {\text{ F}}_{0} }} \times \frac{1}{{{{\varphi }}_{{{\text{P}}0}} }} $$

While this parameter gives a relative size difference of antenna complexes attached to active centers, it does not show the influence of different antenna types. To determine the partitioning between PSIIα and PSIIβ centers, flash fluorescence induction (FFI) analysis was performed after applying a 50 µs single-turnover flash using the 100% relative flash energy on the instrument with the detector sensitivity set at 3%, as outlined in previously published research^32,35,51^. The area over the double normalized curves was integrated using OriginPro (OriginLab, Northampton, MA) and used to generate a semi-log plot consisting of a fast, sigmoidal phase attributed to PSIIα and a slow, linear phase attributed to PSIIβ. The intercept of these two phases reflects the proportion of PSIIβ reaction centers^35,99–101^.

### Analysis of state transitions

State transitions were analyzed using a home-built tabletop 77 K fluorescence spectrometer as previously published, using the FLAME-S spectrophotometer in place of the JAZ-EL200 (Ocean Optics, Largo, FL)^102^. Cell cultures were diluted to 3 µg mL^−1^ Chl *a* with ultrapure water and immediately frozen in liquid N_2_. Chlorophyll were excited using a wavelength of 435 nm and spectra were collected with an integration time of 350 ms and averaged over 16 scans. Peaks for the 77-K fluorescence spectra were determined in OriginPro 2019b using the Multiple Peak Fit tool. Briefly, data were subjected to multiple Gauss fittings in NLFit with Auto Parameter Initialization, six peaks were necessary to account for the area under the spectral envelope.

### Oxygen-evolving complex activity

Photosynthetic oxygen production was measured using a Clark-type oxygen electrode (Oxygraph; Hansatech, UK) at 25 °C as previously described^36^. Oxygen measurements were conducted by exposing 1:1 media diluted cell suspension in modified *Chlorella* media to a 1700-µmol photons· m^−2^ s^−1^ white light after a 5-min dark adaptation phase. High light intensity was chosen in order to ensure light saturation of PSII—the initial rate of oxygen production was measured, normalized to Chl *a*, and used for comparison across culture conditions.

### Statistical analysis

Statistical analyses were performed with GraphPad Prism 7.01 (GraphPad Software, Inc.). Data were analyzed through a one-way Kruskal–Wallis test, comparing each experimental group to that of the control. The family-wise error rate for each figure was maintained at 0.05 using the Holm-Bonferroni method. Statistical significance is indicated numerically through increasing asterisks, where * indicates p ≤ 0.05, ** indicates p ≤ 0.01, *** indicates p ≤ 0.005, and **** indicates p ≤ 0.001. Figures show the means of data (n = 6) and the error bars denote the standard error of the measurement, unless otherwise stated.

## Supplementary Information

Supplementary Figures.

Supplementary Tables.

## Data Availability

All data generated or analyzed during this study are included in this published article and its supplementary information files.
