# Supplementary material for: Excess manganese increases photosynthetic activity via enhanced reducing center and antenna plasticity in Chlorella vulgaris
Source: Sci Rep. 2023 Jul 12;13:11301. doi: 10.1038/s41598-023-35895-x (PMC10338473; doi:10.1038/s41598-023-35895-x)
Supplement: Supplementary file 1 — Supplementary Figures. [file 41598_2023_35895_MOESM1_ESM.pdf]

Excess manganese increases photosynthetic activity via enhanced reducing center and antenna plasticity in *Chlorella vulgaris*

Amanda L. Smythers[1,2], Jessica R. Crislip[1], Danielle R. Slone[1], Brendin B. Flinn[1,] Jeffrey E. Chaffins[1], Kristen A. Camp[1], Eli W. McFeeley[1], and Derrick R.J. Kolling[1]*

[1]Marshall University, Department of Chemistry, Huntington, WV, USA
[2] Current: University of North Carolina at Chapel Hill, Department of Chemistry, Chapel Hill, NC, USA

* **Correspondence**:
Derrick R. J. Kolling, Ph.D.
kolling@marshall.edu

Supplemental Figures

Supplemental figure 1. Immunoblot of the PsbA subunit of PSII. Pellet algal samples were extracted overnight at 4 °C in 4 M urea in PBS, with 5% w/v SDS and 500 mM EDTA. Following clarification, proteins were washed once with extraction buffer following precipitation with chilled 70% v/v ethanol and resuspended in 4 M urea in PBS, 5% SDS. Protein was quantified using a CB-X protein assay. Protein (5.4 ug per lane) was separated via SDS PAGE and blotted onto a PVDF membrane. Proteins were incubated with a 1:10,000 solution of rabbit anti-PsbA (Agrisera, AS10 704) overnight, before rinsing and incubating in a solution of 1:10,000 horseradish peroxidase-conjugated donkey anti-rabbit IgG for 1 he. Protein bands were detected by chemiluminescence.

Supplemental figure 2. The averaged double normalized OJIP curves of 6 biological replicates of each manganese concentration.

Supplemental figure 3. The complementary area over the double normalized OJIP curve. The error bars represent standard error of the mean and statistical differences indicate a difference between the increased manganese and control cultures at one concentration. Significance is denoted by asterisks, where * indicates $p \leq 0.05$, ** indicates $p \leq 0.01$, *** indicates $p \leq 0.001$, and **** indicates $p \leq 0.0001$.

Supplemental figure 4. The averaged S-state traces over 6 biological replicates of each manganese concentration normalized to F0 = 1.

Supplemental figure 5. The averaged FFI traces over 6 biological replicates of each manganese concentration normalized to F0 = 0 and Fm = 1.

Supplemental figure 5. The 77K spectra collected averaged from four biological replicates of each concentration.

Supplemental figure 6. A representative example of a 77 K spectra deconvolution for each manganese concentration. Four biolgical replicates of each concentration were deconvoluted using OriginPro and averaged, as shown in Table 1.

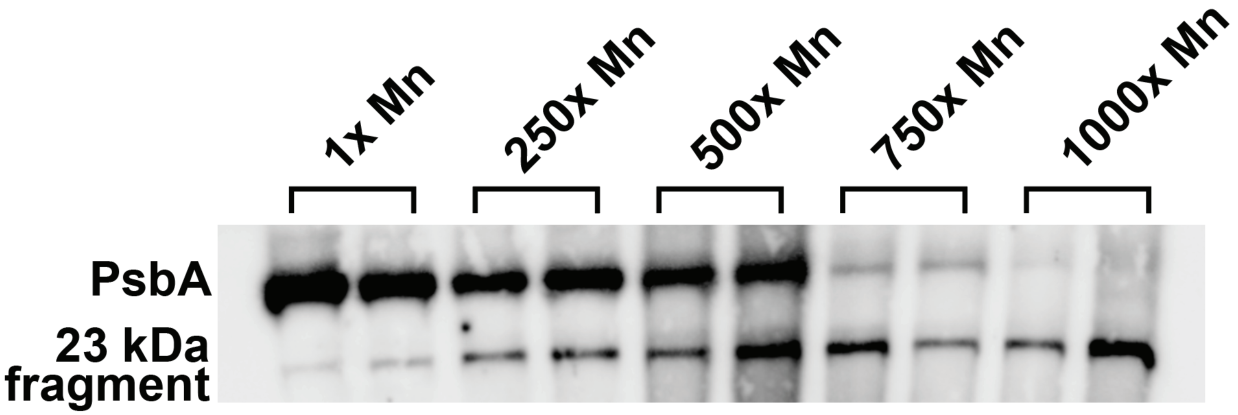

Supplemental figure 1. Immunoblot of the PsbA subunit of PSII. Pellet algal samples were extracted overnight at 4 °C in 4 M urea in PBS, with 5% w/v SDS and 500 mM EDTA. Following clarification, proteins were washed once with extraction buffer following precipitation with chilled 70% v/v ethanol and resuspended in 4 M urea in PBS, 5% SDS. Protein was quantified using a CB-X protein assay. Protein (5.4 ug per lane) was separated via SDS PAGE and blotted onto a PVDF membrane. Proteins were incubated with a 1:10,000 solution of rabbit anti-PsbA (Agrisera, AS10 704) overnight, before rinsing and incubating in a solution of 1:10,000 horseradish peroxidase-conjugated donkey anti-rabbit IgG for 1 he. Protein bands were detected by chemiluminescence.

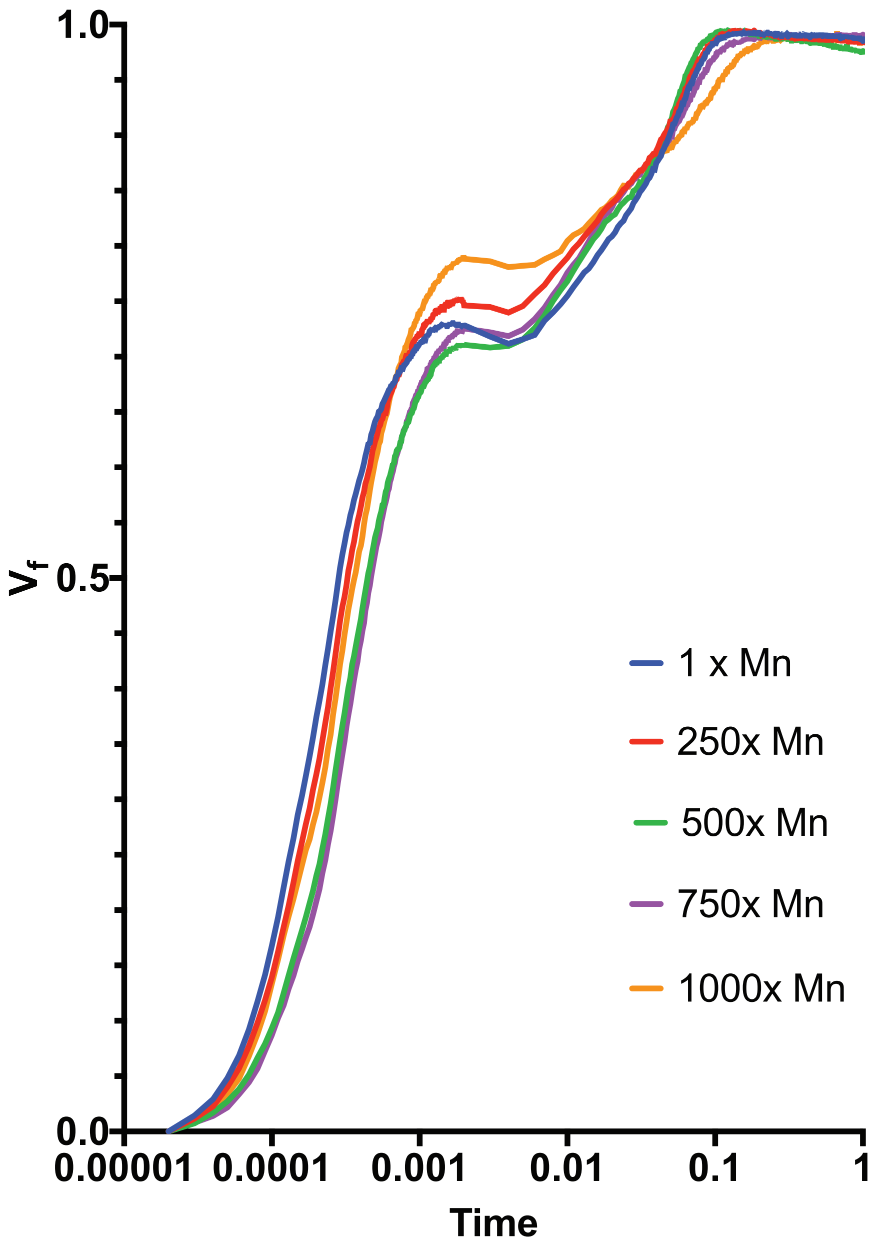

Supplemental figure 2. The averaged double normalized OJIP curves of 6 biological replicates of each manganese concentration.

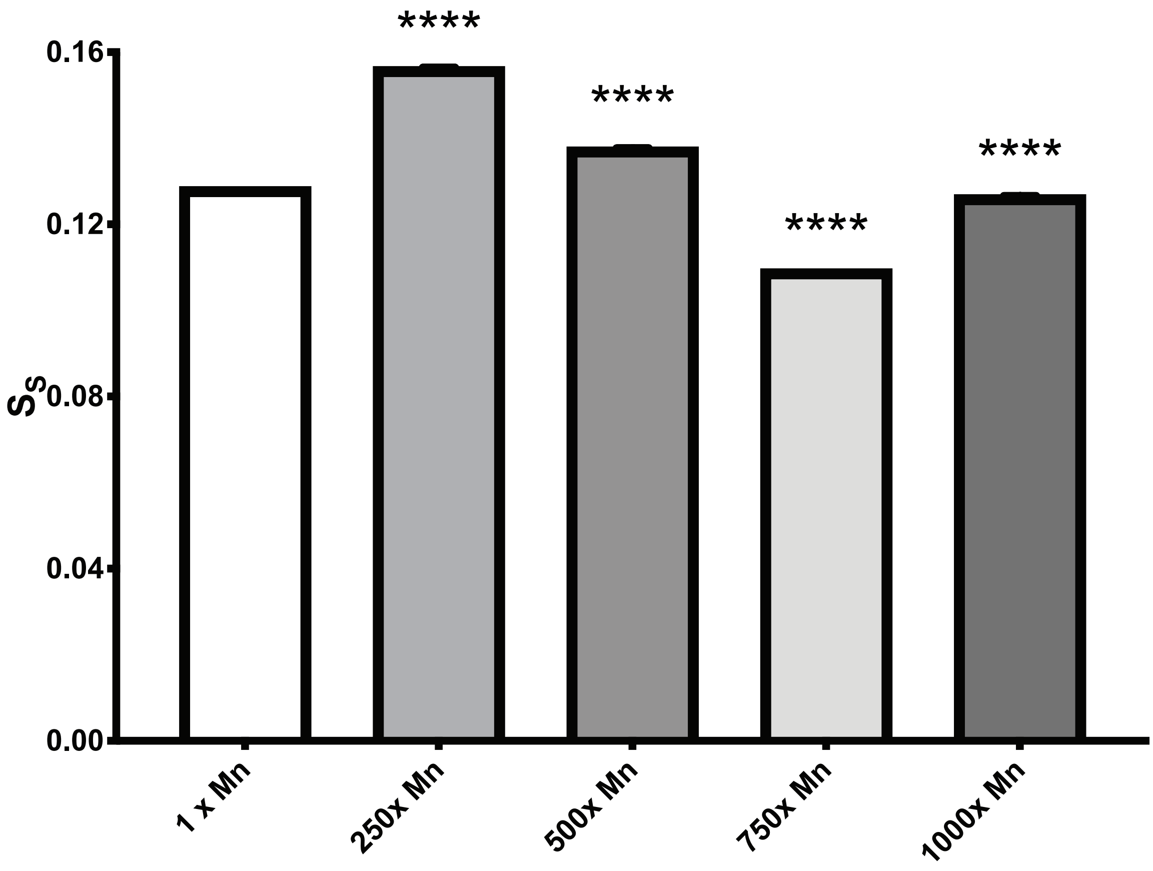

Supplemental figure 3. The complementary area over the double normalized OJIP curve. The error bars represent standard error of the mean and statistical differences indicate a difference between the increased manganese and control cultures at one concentration. Significance is denoted by asterisks, where * indicates $p \leq 0.05$, ** indicates $p \leq 0.01$, *** indicates $p \leq 0.001$, and **** indicates $p \leq 0.0001$.

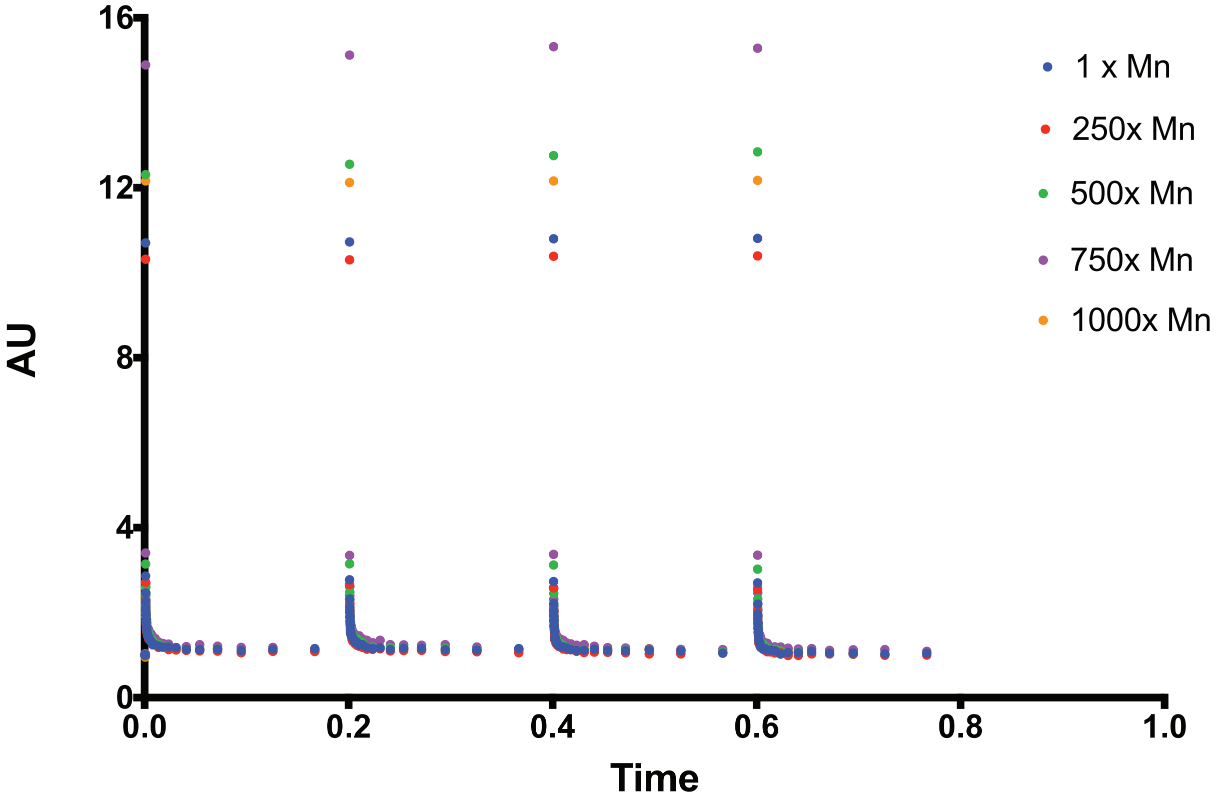

Supplemental figure 4. The averaged S-state traces over 6 biological replicates of each manganese concentration normalized to F0 = 1.

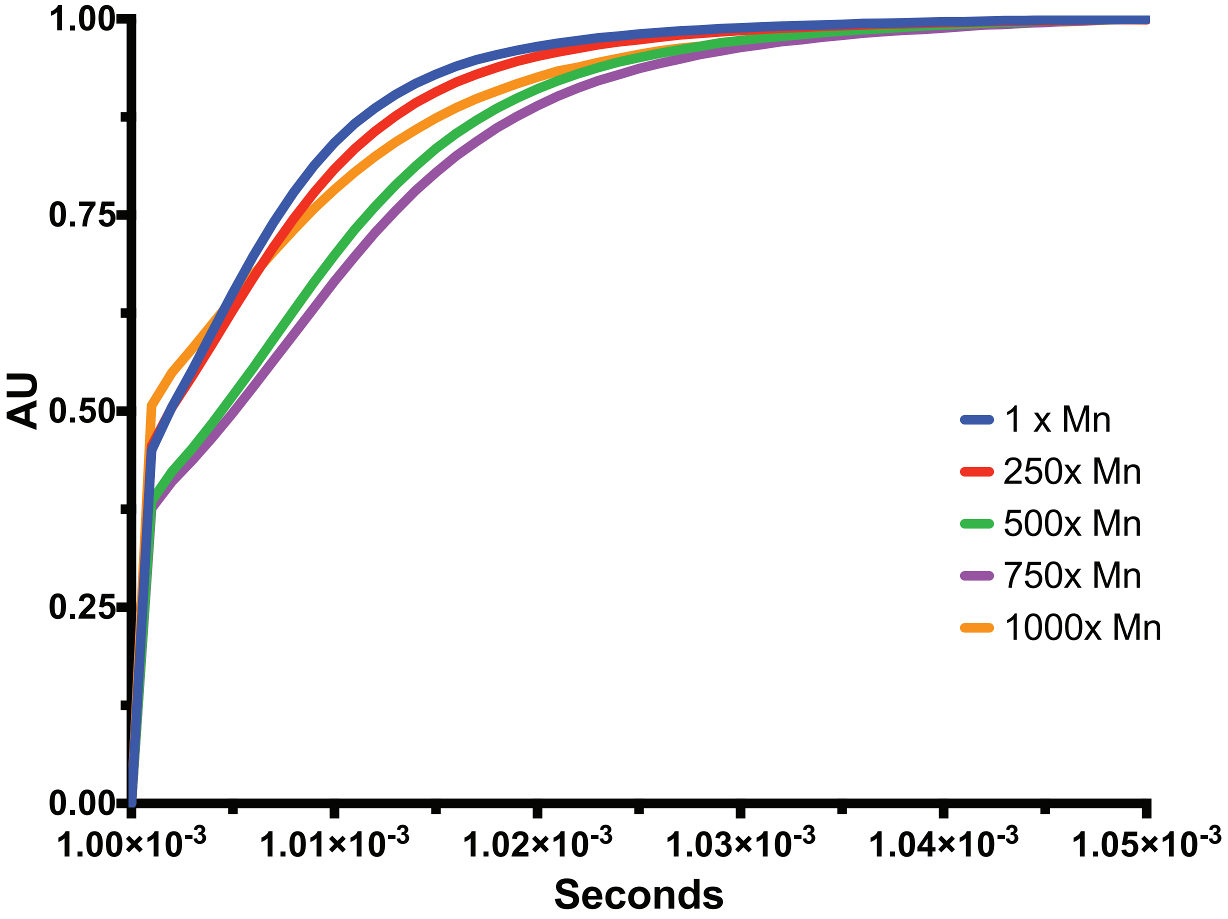

Supplemental figure 5. The averaged FFI traces over 6 biological replicates of each manganese concentration normalized to F0 = 0 and Fm = 1.

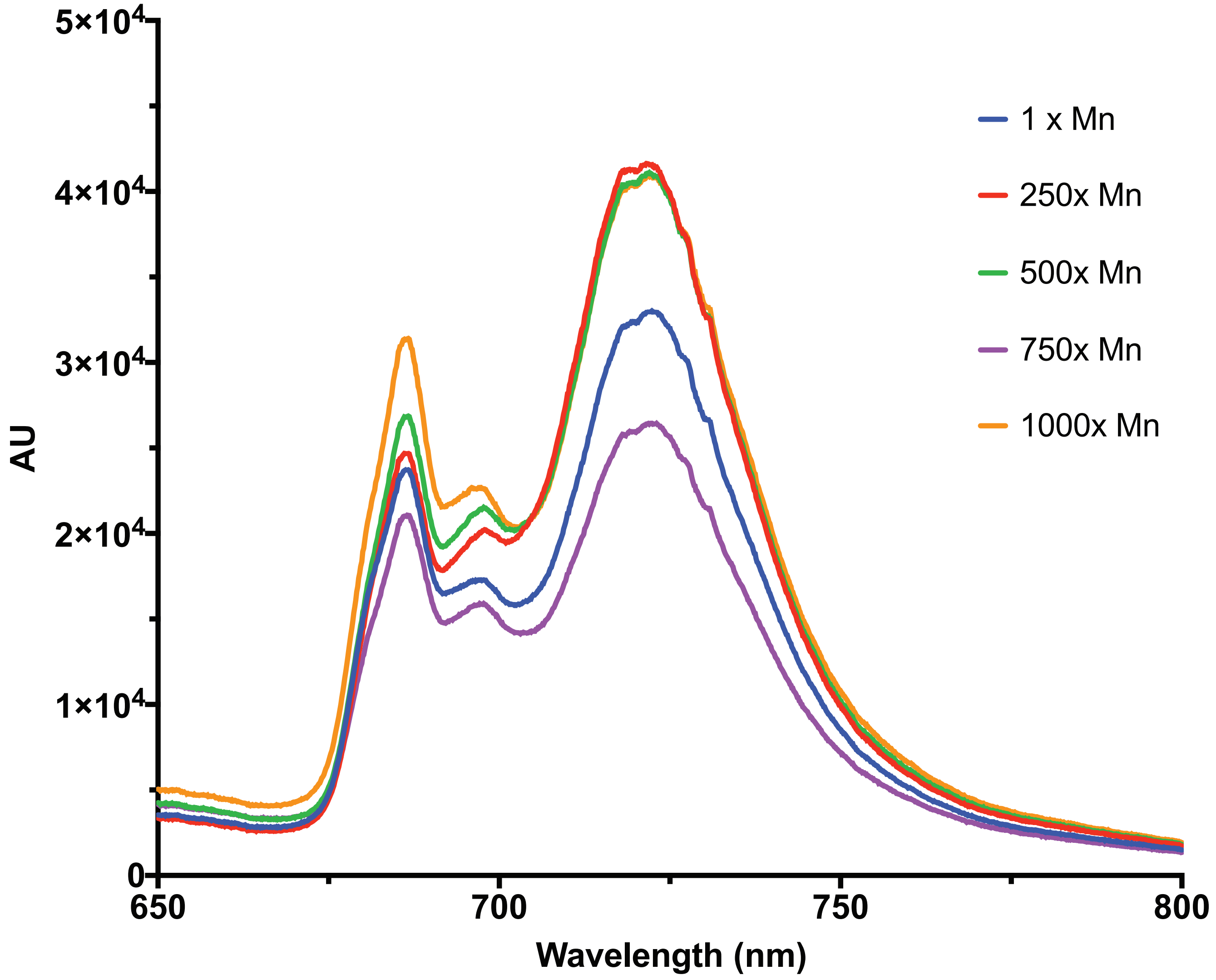

Supplemental figure 6. The 77K spectra collected averaged from four biological replicates of each concentration.

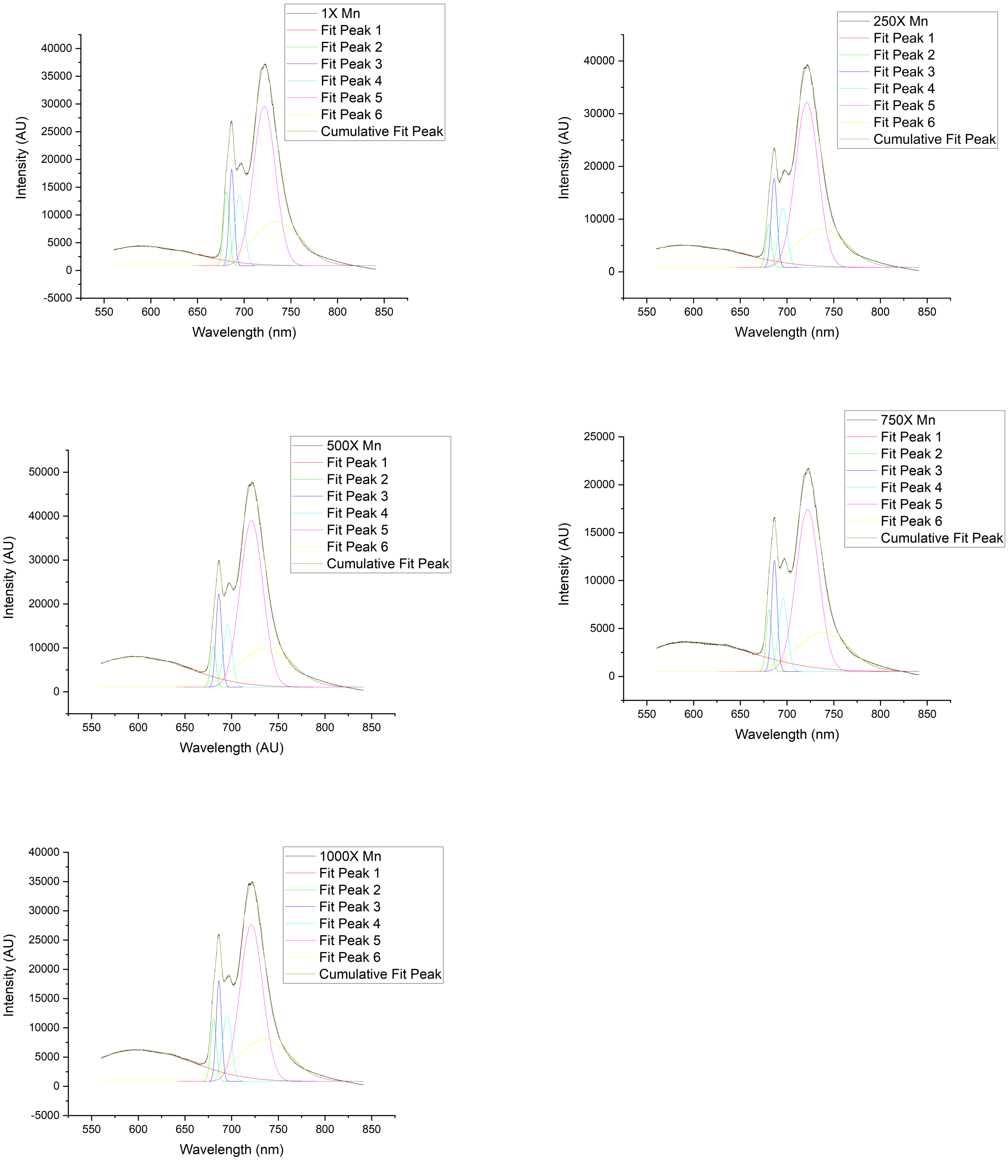

Supplemental figure 7. A representative example of a 77 K spectra deconvolution for each manganese concentration. Four biolgical replicates of each concentration were deconvoluted using OriginPro and averaged, as shown in Table 1.
